# Cumulative Environmental Vulnerability Assessment in the Area of Influence of the Pecém Port Industrial Complex (Ceará, Brazil): A Spatial Analysis

**DOI:** 10.3390/ijerph18052404

**Published:** 2021-03-01

**Authors:** Norberto Santos-Junior, Jose Ueleres Braga, Elvira Maria Godinho de Seixas Maciel

**Affiliations:** 1 Programa de Pós-Graduação em Saude Publica e Meio Ambiente, Escola Nacional de Saude Pública—FIOCRUZ 1, Rio de Janeiro 21041-210, Brazil; norberto1808@gmail.com; 2 Departamento de Epidemiologia e Metodos Quantitativos em Saude, Escola Nacional de Saude Pública—FIOCRUZ 2, Rio de Janeiro 21041-210, Brazil; 3 Departamento de Epidemiologia, Instituto de Medicina Social, Universidade do estado do Rio de Janeiro, Rio de Janeiro 20550-013, Brazil

**Keywords:** environmental health, environmental pollutants, air pollution, spatial analysis

## Abstract

The municipalities of Caucaia and São Gonçalo do Amarante are located in the metropolitan region of Fortaleza (CE) and are home to the Pecém Port Industrial Complex (PPIC). We know that economic development is not necessarily related to improvements in the quality of life of the population. Furthermore, the bonuses and burdens of this particular installation can occur unevenly. This study aimed to assess the cumulative environmental vulnerability of these municipalities. We used the cumulative environmental vulnerability assessment methodology to assess the population’s degree of vulnerability, considering census sectors as a spatial unit. This approach combines three indices: environmental risk index, social vulnerability index, and health index. Finally, we calculated the arithmetic mean of each indicator in each census sector. We built choropleth maps to assess the spatial distribution of environmental vulnerability. We found that many maps demonstrated high cumulative environmental vulnerability census sectors around the PPIC, while the Caucaia, located downtown, exhibited a substantial majority of the low cumulative environmental vulnerability census sectors. The district of Guararu, in Caucaia, was notable for having proportionally more census sectors with high health index values. Environmental vulnerability was heterogeneously distributed, and the most impoverished areas are also the most vulnerable.

## 1. Introduction

In 2002, in São Gonçalo do Amarante (SGA), bordering Caucaia (CAU), the Port of Pecém was inaugurated, and this port was more extensive than the Port of Mucuripe (one of the main coastal cargo handling shipping ports in Brazil, the first landing place for Europeans in the current Brazilian territory), located in Fortaleza. Soon after, efforts began to install an industrial complex nearby. Thus, the Pecém Port Industrial Complex emerged, situated in both CAU and SGA, and occupying an area of approximately 13,337 hectares. Its main productive activities are the production of cement and steel, utilising three thermoelectric plants, and two conveyor belts, for inputs.

Since installation of the PPIC, its host municipalities have transformed their demographics and socioeconomic characteristics. From 1991 to 2010, the population of Caucaia increased from 167,271 to 325,441 inhabitants, whereas the population of SGA increased from 29,457 inhabitants to 43,890 [1]. Although the latter municipality has demographically increased less than its neighbour, the change in its Human Development Index (HDI) was more marked, so that it doubled, from 0.325 to 0.665, while Caucaia increased from 0.411 to 0.682 [2]. Although the HDI of SGA has increased more than that of the municipality of Caucaia, it is worth considering that these have not yet reached a high level of human development (values above 0.700). It is also worth mentioning that only the one related to income was responsible for the change of index’s change.

Enterprises like the PPIC arise with the expectation of generating income and jobs; however, in many cases, they also affect the conditions of the health-disease process due to production. The making of cement and steel, the three thermoelectric plants, and two conveyor belt could potentially lead to contamination of soil, water resources, the atmosphere, as well as potential risks to the health and living conditions of the populations living in the vicinity, as toxic substances and compounds, are released into the environment and can spread over large areas, entering the food chain and affecting human health. Furthermore, there are often no mitigation measures or adequate planning to deal with the deleterious effects arising from such enterprises [3]. The economic development of underdeveloped countries is usually unbalanced, where a minority of society receives the majority of the bonus, and another proportion focuses on the burden, as noted by the Brazilian Ministry of the Environment (2014) [4].

In the case of the PPIC, population and economic growth were accompanied by undesirable transformations, including insufficient services offered, slums, occupation of protected areas, sexual exploitation of minors, early sexual initiation, increased consumption of illicit drugs, and increased crime rates [1]. Indeed, the municipalities of CAU and SGA are experiencing a moment of emergence of deepening of social problems. In the logic of Adeola and Picou, the existence of an unequal social structure shapes environmental injustice. These authors understand environmental injustice as a condition in which specific groups, due to ethnicity, race, income, or nationality, are deprived of their environmental rights or affected in this sense through policies and actions applied unequally. However, deprivation is not limited to access to an ecologically balanced environment, but also affects the population’s livelihood [5]. From an anti-positivist perspective, environmental injustice is the simultaneity of both endogenous and exogenous processes that operate in different locations, with different intensities. These authors also states that environmental injustice, from a non-deterministic perspective, would be the coincidence of physical, material, institutional, political, social, and cultural risks.

To overcome the inequities caused or deepened by human action, the movements and concept of environmental justice emerged in the 1970s. In Corburn’s conception [5]. environmental justice is a tool for better understanding and action in the face of injustice in this field, targeting not only decision-makers but also civil society. Environmental justice, in addition to addressing the physical space where people live and perform their tasks, also addresses the political distribution in the decision-making process and opportunities given to interested parties to manifest themselves [4]. This description is paralleled by the Environmental Protection Agency of the United States (2012) [6], which states that environmental justice is the “fair treatment and significant involvement of all people, regardless of race, colour, national origin, or income, concerning development, implementation, inspection and application of environmental laws, regulations and policies”. Soon Adola and Picou [7] followed this reasoning, stating that environmental justice will only be achieved when everyone enjoys the same protection from environmental and health risks and equal access to the decision-making process, more specifically regarding the environment. For Sabapathy [8] today, environmental justice can be considered one of the principles of social justice, addressing the disproportionate distribution of exposure to pollutants in specific groups of a society by demographic and socioeconomic characteristics.

Exposure to physical, chemical, or biological environmental risks interacts with social risks in a given community [9], which is reflected in their health, and is called vulnerability. The concept of vulnerability allows us to work with more than one risk factor at the same time, and thus observe their interactions. DeFur et al. [10] defined vulnerability as an individual or community’s ability to react to the risk to which they are exposed. This statement agrees with Azevedo Penna’s [9] understanding, which defines vulnerability as the offer and quality of services and infrastructure for the protection of communities. At the same time, Penna [11] states that the concept of vulnerability acts as a mechanism that facilitates the debate on policies to combat inequality in a broad way. The simultaneity of environmental, social, and health risks affecting a community or individual is defined by Huang and London [12] as cumulative environmental vulnerability. From this conception, they developed a tool that addresses the nuances of risks or impacts that accumulate in communities. In this way, cumulative environmental vulnerability operates in three dimensions: the cumulative environmental risk index, social vulnerability, and the health index. Supporters of environmental justice understand that academia and regulatory agencies should more often address cumulative impacts to synthesise and complement current models of impact and risk assessment that operate in a compartmentalised manner [13]. Solomon et al. [14] point out the cumulative consequences of disparities in health, with roots in environmental and social factors, significantly uneven environmental exposures, biological and physiological factors that influence the effects of environmental exposures, and social vulnerability at the individual or community level. Alexeeff et al. [15] defined cumulative impact as, briefly, exposures and their impacts on public health and the environment in a geographic area. These exposures of different compositions, sources, and data analyses must also consider the local socioeconomic characteristics. This study aims to assess the cumulative environmental vulnerability and its heterogeneity in the area covered by the PPIC, describing the spatial distribution of the accumulated environmental vulnerability in the municipalities of Caucaia and Sao Gonçalo do Amarante and assess the vulnerability indices of the environmental, social, and health dimensions.

## 2. Materials and Methods

The methodological approach of this study was based on the cumulative environmental vulnerability and environmental justice utilized in California’s San Joaquin Valley Project [12]. The epidemiological design was an ecologic study of multiple groups, whose unit of analysis was the census sectors in the municipalities of Caucaia and São Gonçalo do Amarante. Three components compose the cumulative environmental vulnerability assessment approach: (i) the cumulative environmental risk index—CERI; (ii) the social vulnerability index—SVI; and (iii) the health index—HI. These indices resulted from an arithmetic average of normalised values from a set of specific indicators for each component index, CERI, SVI, or HI. The CEVA interpretation considers the levels of CEVI and SVI (low, intermediate, and high). These socio-environmental vulnerability levels are confronted with HI so that health can indicate the effects of this vulnerability.

The cumulative environmental risk index (CERI) consists of six indicators: (i) the location of the thermoelectric plant (TEP); (ii) the location of the Pecem Siderugic Company (PSC); (iii) cement plants; and (iv) transfer towers (TT) of the conveyor belts (CT) for coal and ore to the TEP and the location of the PSC; (v) contamination of waters; and (vi) atmospheric contamination.

The first four indicators together make up the index of proximity to the source of pollutant emissions. This index, which had the function of an indicator, was calculated by drawing buffers of 1.6 km radius around these locations and subsequently overlapping them using the QGIS 2.18 software (Free Software Foundation, Inc., Boston, MA, USA). From this, the percentage of areas covered by the buffers was calculated, which indicates how close the populations are to at least one of the pollutant-emitting sources. This index value ranged from 0 to 1, so that census sectors with a value of 1 are those that were entirely covered by the buffers, while 0 represents census sectors that were not within 1.6 km of any source of pollutant emission.

We used secondary data for the years 2015 and 2016, based on the presence or absence of total coliforms to calculate a water contamination indicator. This indicator had dichotomous values, 0 for the absence of total coliforms and 1 for the presence of total coliforms. The collection points were georeferenced and evaluated if they were inside the census sector and classified according to the presence of total coliforms.

We calculated the atmospheric contamination indicator using the atmospheric data of particulate matter smaller than 2.5 µm, nitrogen dioxide, carbon monoxide, and ozone. We used the MERRA data set (Modern-Era Retrospective analysis for Research and Applications, Era Modenas de Retrospective Analyzes for Research and Applications) from NASA between 2011 and 2015. We then calculated the weighted averages of the magnitude of pollutants in each census sector and in each month of the study period. Finally, the arithmetic average and atmospheric contamination in each census sector were calculated over the study period.

The arithmetic average of the values for each indicator in the census sectors was calculated and used as normalised data to measure the degree of cumulative environmental risk on a scale of 0 to 1, where 0 indicates the minimum risk and 1 indicates the maximum risk.

The formula used to calculate CERI was:

(1) $$CERI_{i}=\frac{\sum_{j=1}^{n}v_{ij}}{n}$$

and:

(2) $$CERI_{i\;norm}=\frac{CERI_{i}}{CERI_{i\;max}}$$

where *v_ij_* is equivalent to the sum of the values of the index of local pollutant emission source + indicator of water contamination indicator + atmospheric contamination indicator; *CERI_i_* = CERI not normalised; *n* = number of index component indicators; (*CERI_imax_*) = highest *CERI_i_* value obtained from *CERI_i_* among all census sectors; *CERI_inorm_* is the normalised CERI.

The Social Vulnerability Index (SVI) was composed of the following indicators: (i) the percentage of people under five or over 60 years of age; (ii) location of health services; (iii) presence of ethnic minorities; (iv) the percentage of people in poverty; and (v) the percentage of people over 15 years of age who are illiterate.

The indicator of the location of services was estimated using the georeferencing of health establishments in such a way that 1.6 km radius buffers were drawn around them. Thus, the percentage of the area of the census sectors that were not covered by buffers was converted to decimals ranging from 0 to 1.

We estimated the other indicators using 2010 census data, where the proportion of people with the characteristics described in the total population of the census sectors was calculated. Subsequently, the values were normalised, ranging from 0 to 1. Finally, we calculated the indicator as the arithmetic average for each census sector; the closer to 1, the greater the social vulnerability.

The health index was composed of indicators of low birth weight, potential years of life lost before 65 years of age, and rate of hospitalisation for asthma in people under 20 years of age. We calculated the low-birth-weight indicator using data from the Brazilian Newborn Registration System of the municipalities of Caucaia and São Gonçalo do Amarante, referencing those according to their place of residence in the census sectors. Therefore, in each of these locations, the rate of low birth weight was calculated, considering a low birth weight of 2.5 kg and live births since the mother lives in one of the municipalities surveyed. We calculated the Potential Years of Life Lost (PYLL) indicator before the age of 65 using the Death Registration System (SIM) data for residents of the municipalities between the years 2011 to 2016, and the average PYLL before 65 years of age for each census sector. Finally, based on the hospitalisation records system data, we calculated the rate of hospitalisations for asthma among children under 20 years of age living in these municipalities. We normalised the indicator values, the closer to 1, the worse the health status of a community and 0 reflects the opposite. 

CERI and SVI indexes were categorised into three levels based on terciles: low, medium, and high. Later on, it was possible to measure the socio-environmental vulnerability of the census sectors, where these two dimensions interacted and nine classifications: low SVI and high CERI; median SVI and high CERI; high SVI and high CERI; low SVI and median CERI; median SVI and median CERI; high SVI and average CERI; low SVI and low CERI; median SVI and low CERI and high SVI and low CERI. The health index was categorised into four groups: low, medium, high, and very high, so that they were separated into quartiles of the index values.

**Figure 1:**
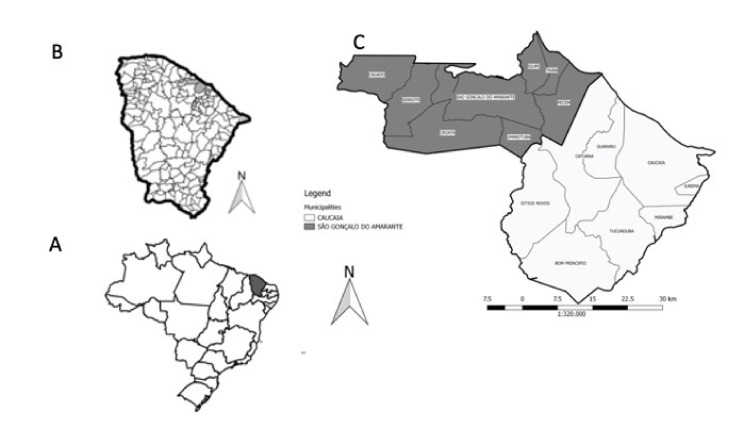
Maps of Brazilian states (**A**), Municipalities of State of Ceará (**B**) and Districts of the municipalities of São Gonçalo do Amarante and Caucaia (**C**).

The study area comprised the municipalities of São Gonçalo do Amarante (3°36′21″ S; 38°58′08″ W) and Caucaia (03°43′58″ S; 38°39′21″ W) located west of the capital of Ceará, Fortaleza. The units of analysis were 68 census sectors of SGA and 414 of Caucaia and its districts (Figure 1).

The data used in this study were made available by the Ceará State Health Department without the identification of the records and the project was approved by the Ethics Committee of the National School of Public Health of FIOCRUZ on 16 August 2018 CAEE 94976718.3.0000.5240.

## 3. Results

### 3.1. Cumulative Environmental Risk Index

The five highest CERIs were found in the district of Pecém in São Gonçalo do Amarante. In contrast, two of the five lowest CERI values occurred in the district of Croatá and the remaining three in Cágado, all belonging to São Gonçalo do Amarante. There were approximately 434 census sectors with the same CERI value that lay between the maximum and minimum values. Among the 12 census sectors in Pecém, 11 had high CERI, and only 1 had low CERI. However, among the census sectors in Catuana that border the district of Pecém, all showed high CERI.

The indicators of proximity to polluting sources pointed to only a few census sectors, belonging to the districts of Catuana and Pecém, Caucaia, and São Gonçalo do Amarante, so that all census sectors that showed high proximity to polluting sources were also among those with high CERI.

The lowest value for the indicator of atmospheric pollution occurred in a census sector in the district of Serrote, São Gonçalo do Amarante. It was possible to identify census sectors classified as experiencing low pollution only in the census mentioned above, in addition to Cágado and Croatá. In contrast, of the entire study area, 463 census sectors exhibited the maximum value of atmospheric pollution, so that some districts were composed entirely of census sectors with the maximum value. Only Cágado, Serrote, and Bom Princípio did not have at least one census sector that exhibited this degree of air pollution, while Tucunduba and Croatá had only a few census sectors in this condition. Tortoise, in particular, kept all its census areas in the low atmospheric pollution range. One of the major factors that helps explain this peculiarity in Cágado is because this is the most outlying district of Pecém. However, this factor alone does not explain this difference, because some census sectors in Croatá were closer to Pecém than Serrote, but still had less air pollution.

**Figure 2:**
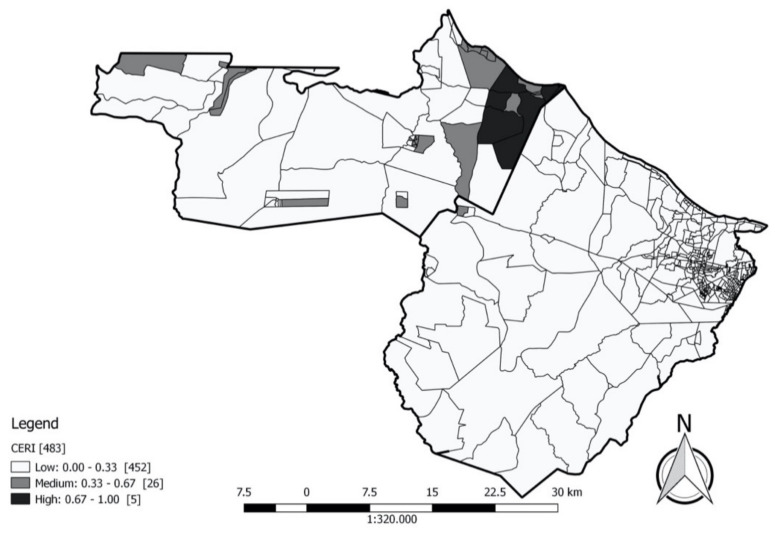
Map of Cumulative Environmental Risk Index of the census sectors in the municipalities of São Gonçalo do Amarante (left) and Caucaia (right), Ceará.

Water contamination during the study period was not detected in 454 census sectors; however, there was continuity in census sectors where total coliforms were detected between the districts of Taíba and Pecém. According to CEARÁ [1], the arrival of workers with better education and better placement in the labour market intensifies the pressure for the offer of housing, services, and infrastructure and this contrasts with the reality of native populations that did not have access to these resources. Specifically, in the SGA Headquarters District, the same census sectors with high water contamination were the same with low SVI. It is noteworthy that although SGA is the municipality that houses the majority of PPIC’s enterprises, it is mostly rural if we count the number of census sectors of each type, unlike Caucaia, which is primarily urban. Therefore, although population growth was more remarkable in Caucaia, the challenges of improving urban infrastructure are more concentrated in SGA, and this includes the headquarters district, which will be the reference in this regard for all other municipalities in this regard (Figure 2).

### 3.2. Social Vulnerability Index

The lowest SVI occurred in the census sector belonging to the district of Jurema. In contrast, the highest value of the index occurred in a census sector in the district of Bom Princípio, in Caucaia, and the census sector with a median SVI was in the district of Jurema (Caucaia). At the extremes, there was a discrepancy in the type of census sector so that the 11 highest SVIs were associated with rural areas, while the 74 lowest SVIs were urban. Among the 161 census sectors considered as having a low SVI, 150 belong to either the District Headquarters of Caucaia or Jurema, districts bordering each other, and which are the only districts adjacent to Fortaleza. According to CEARÁ [1], the municipality of Caucaia has an old economic relationship with Fortaleza and a transport network that reflects this. Thus, it can be deduced that less socially vulnerable populations in our study area tend to seek housing in places where the offer of services, income, and jobs is more generous.

The highest proportion of poor people occurred in a census tract within the rural area of the district of Bom Princípio in Caucaia. In contrast, the lowest proportion of poor people was in an urban census sector in the District Headquarters of Caucaia. The census sector having an average position belongs to the district of Jurema. Both the district of Sede of Caucaia and Jurema were characterised by having 139 of the 161 census sectors that make up the one third of the low proportion of poverty. All census sectors in Mirambé were in the high proportion of low-income families. This contradicts, in part, the logic that populations closer to capital cities or large urban centres benefit economically from this proximity because Mirambé is the nearest district to Fortaleza among those who did not border the capital of Ceará. Even in Jurema and in the District Headquarters of Caucaia, there were census sectors classified as low-income, including all census sectors in the district headquarters of Caucaia that border Mirambé. It must be considered that locations where, in theory, there is a larger supply of services, infrastructure, and employment opportunities, will not necessarily reach everyone living there and therefore will not necessarily attract only those who, in theory, can afford the highest living costs associated with these localities, albeit through subnormal clusters.

Among the 161 census sectors considered as having a high proportion of low-income families, 116 were among those with high SVI. Analysing vulnerability from a social point of view requires a series of factors to be examined that go beyond income. However, since we are using indicators such as education and race, we must consider the possibility that poverty is already partly a result of racism and low education, as well as high social vulnerability.

**Figure 3:**
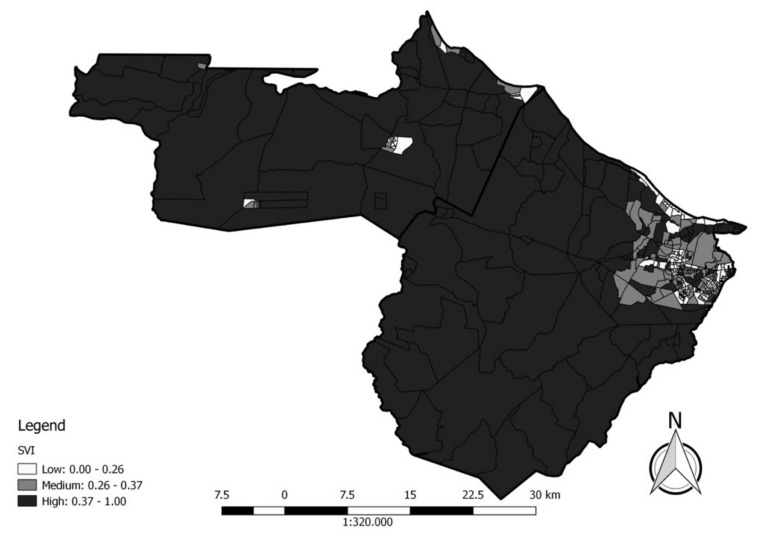
Map of Social Vulnerability Index of the census sectors in the municipalities of São Gonçalo do Amarante (left) and Caucaia (right), Ceará.

The lowest proportion of illiteracy among those over 15 years of age occurred in a census sector in the district Sede de Caucaia. However, the median value of the proportion of illiteracy, and the highest proportion of illiteracy in this age group, also occurred in this district, where three census sectors exhibited the maximum value, along with a census sector in the SGA Headquarters District. Most of the census sectors with a low or medium proportion of illiterates over 15 years of age were found in the headquarters districts of Caucaia, São Gonçalo do Amarante, and Jurema; thus, almost all other census sectors were classified as containing a high proportion of illiterates (Figure 3). The only census sectors with a low proportion of illiterates outside the districts mentioned above were in the districts of Pecém, and São Gonçalo do Amarante. Among those considered as having a high proportion of illiterate, 60 were rural and 88 urban. In contrast, among those with a low proportion of illiterates, four were rural, while 144 were urban. Unlike poverty, where high rates are not usually ubiquitous in the districts, with heterogeneity observed regarding the proportion of low-income families, illiteracy was differentiated by the fact that there were several districts where all census sectors exhibited a high proportion of illiteracy among those over 15 (Figure 3). As a rule, low education is accompanied by participation in less well-paid occupations.

### 3.3. Health Index

The two largest HI were found in the District Headquarters of Caucaia, more specifically in Parque Soledade and Parque Leblon. However, 71 census sectors scored zero, followed by a census sector in the district headquarters of Caucaia, in the neighbourhood of Itapoã. Considering only those census sectors where a zero-value occurred, the median would have been for a census sector in the district headquarters of Caucaia, in the neighbourhood of Guagiru. However, if we exclude census sectors exhibiting a zero value, the median will be between two census sectors, both from the district headquarters of Caucaia, but in the neighbourhoods of Tabuba and Conjunto Metropole.

**Figure 4:**
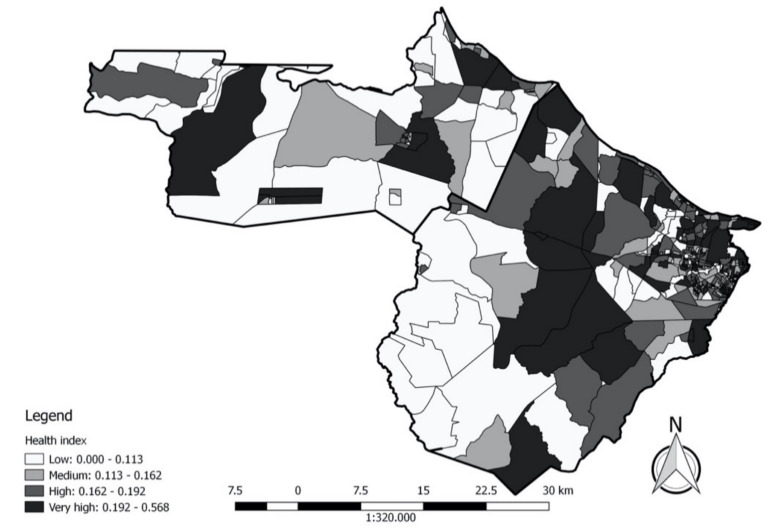
Map of Health Index of the census sectors in the municipalities of São Gonçalo do Amarante (left) and Caucaia (right), Ceará.

The lowest mean PYLL up to 65 years was found in nine census sectors, six of which belong to the District Headquarters of Caucaia and one to Jurema, Bom Principio, and Pecém. In contrast, the highest average of PYLL up to 65 years occurred in five census sectors, and two belong to the district of Serrote and one to Croatá, Guararu, and Distrito Sede de Caucaia. The district of Guararu was the only district to be filled by census sectors having a high average PYLL. In contrast, the only districts that did not have a census sector with a high average PYLL were Bom Princípio, Umarituba, and Siupé (Figure 4).

The lowest rate of hospitalisation for asthma in children under 20 years of age occurred in 262 census sectors, of which 201 were located in Caucaia and 61 were located in SGA, all with a zero-value. This value was also the median value. In contrast, the highest rate was in a census sector located in the District Headquarters of Caucaia; the six highest rates were in census sectors in that district. The District Headquarters of Caucaia, District Headquarters of SGA, Mirambé, Jurema, and Sítios Novos were the only districts to not have census sectors with low rates of hospitalisation for asthma.

The lowest proportion of low birth weight occurred in 92 census sectors, of which 79 were located in Caucaia and 13 were located in SGA. In contrast, the largest proportion occurred in the census sector in the District Headquarters of Caucaia, more specifically in the neighbourhood of Parque Soledade. The census sector that occupied an average position was located in the district Sede de Caucaia, more specifically in the neighbourhood of Padre Julio Maria. Among the coastal census sectors, only five had a low proportion of low birth weight (Figure 4).

### 3.4. Cumulative Environmental Vulnerability Classification

**Figure 5:**
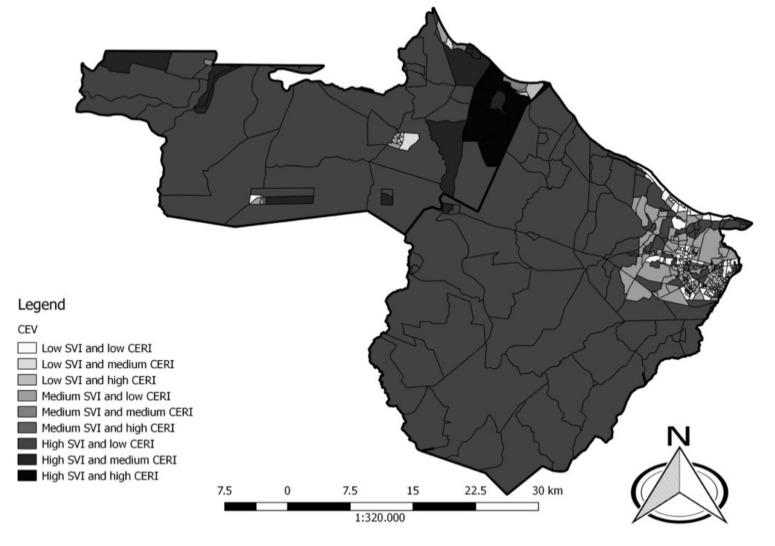
Map of cumulative environmental vulnerability classification of the census sectors in the municipalities of São Gonçalo do Amarante (left) and Caucaia (right), Ceará.

We classified all census sectors by cumulative environmental vulnerability, and four census sectors were identified as having a high CERI and high SVI and these were restricted to the district of Pecém. The opposite, low CERI and low SVI, occurred in some census sectors of the district Sede de Caucaia, Jurema, and Croatá, with 0.71, 0.78, and 1, respectively. Of these, four were considered as rural and all belonged to the District Sede de Caucaia. The districts of Sítios Novos, Bom Princípio, Tucunduba, Mirambé, and Guararu in Caucaia, were characterised as having all their census sectors classified as high SVI and low CERI. In São Gonçalo do Amarante, the only district where this occurred was Siupé (Figure 5).

## 4. Discussion

The main finding of this study is that environmental vulnerability was heterogeneously distributed in the study area. Very high levels of cumulative environmental vulnerability were essentially restricted to the area of influence of the PPIC, and the district of Pecém in São Gonçalo do Amarante. The cumulative environmental risk, assessed from 2011 to 2016 in Caucaia and São Gonçalo do Amarante, was markedly higher in the PPIC region and, mainly, in the Pecém district in São Gonçalo do Amarante. Social vulnerability is much more significant in rural areas of these two municipalities. Only the headquarters of municipalities and coastal regions had a low level of social vulnerability. The distribution of the health index was also relatively heterogeneous, and it was lower in rural areas, and far from the headquarters of the municipalities studied.

The notion of vulnerability is found in numerous studies of the impact of natural events, or changes induced by economic activities, on human health. It must be understood in the environmental (climate change, the occurrence of extreme weather events and natural disasters), social (construction and operation of economic enterprises of different nature, keeping in common the possibility of interfering in morbidity and mortality patterns), and biological domains [16].

Vulnerability was defined as the state of risk of an individual or population. The study sought cumulative environmental risk in a location in the Greater Fortaleza, Metropolitan Region of the capital of the state of Ceará, Brazil. This study site was chosen because of the implementation, initiated in the mid-90s, of the Pecém Port Industrial Complex, considering that the performance of new economic activities in territories with specific geographical, demographic, and social characteristics usually brings harmful consequences to population well-being [17]. In the present case, the development of the port and of steel, thermoelectric, and other industrial activities, appears to have also led to significant economic transformations (variations of GDP and its repercussions), and social transformations (variations in the HDI, migratory flows, demographically high-density areas, expropriation, and resettlement of population groups). Thus, considered in socioeconomic and environmental terms, vulnerability is related to notions of sustainability and justice [16].

The index used herein, the cumulative environmental vulnerability index, was proposed by Huang and London (2012) [12] in a study carried out in the São Joaquim Valley region (California, USA). There are commonalities and points of difference between the municipalities of Ceará and the San Joaquin Valley region. The first and perhaps most relevant common point is the notion of “environmental justice” or the need, also in the case of Caucaia and SGA, to limit the proximity of places of habitation and the sources emission and dispersion of pollutants. Furthermore, by the social and health indicators of residents, one can infer a greater socio-environmental vulnerability in the municipalities of Ceará. This calculation should confer, at different levels of management, degrees of responsibility for potential risks to the health and well-being of residents.

### 4.1. Environmental Risk and Social Conditions

The CEVA index allows, with the application of spatial analysis techniques, identification of areas in which environmental risks posed by the activities developed, result in low social and economic indices, in the absence of public health policies. Monitoring and mitigation measures applied to areas with greater vulnerability and less capacity to adapt to social changes (influx of people in search of jobs and income, resettlement, lack of sufficient infrastructure services and assistance), exposure to contaminants, and recovery in the event of environmental disasters and their consequences. Hence, development of an index composed of the environmental, social, and political dimensions that identifies areas should be prioritised to receive investments capable of minimising risks, preventing and reducing damage [12], and stimulating resilience.

Considering the nature of the activities for which the PPIC is intended, including refinery, steel-making, metallurgy, cement production, energy production from thermoelectric plants, we believe it is appropriate to seek instruments that allow us to prioritise areas for infrastructure investments to minimise the risk of disasters, including monitoring by governmental inspection bodies in the three instances (federal, state and municipal governments), and planning immediate actions in the case of accidents that could not be avoided. In addition, monitoring the health status of the population and the mortality profile, with particular attention to causes related to the operation of the enterprise, is of great importance. The health service network must comply with the regulations in law throughout the territory, plus training and qualification for professionals related to the specificities of the area in question.

### 4.2. Socio Vulnerability and Its Consequence on Health 

There are health conditions that, by increasing individual vulnerability in general, also increase the specific vulnerability in the case of exposure to certain contaminants, leading to the demand for specialised care of a higher level and complexity. Intersectionality is essential, and education is a crucial strategy for increasing the risk perception and taking advantage of new opportunities. In the case of a large port, the change in the size and weight of vehicles changes significantly, and the adaptation of everyone, especially children, the elderly, and the disabled to the new traffic pattern must be continuously encouraged as well as investment in transformation and maintenance of the road network, especially of roads close to the most densely populated areas. Education can also contribute to the prevention of abuse of alcohol and legal and illicit drugs.

Another factor to be considered is the increase in prostitution and the prevention of sexually transmitted diseases (STDs) must be reinforced. In Brazil in general, as in the case of the PPIC, the implementation of a large enterprise brought about an increase in violence, use of illicit drugs, prostitution, and sexually transmitted diseases (data not shown). A related problem is that adolescent living in the territory are also affected, and health and education actions must also be devoted to the theme of family planning and unwanted pregnancies.

Despite the limitations of the index developed by Huang and London [12] (or features of the index), it offers several advantages in the prediction and prevention of environmental accidents, instrumentalising government agencies to deal with the highly vulnerable society. In Brazil, aspects such as drug trafficking, increased use of illicit drugs, alcoholism, violence (including domestic), sexual exploitation of adolescents and children, human trafficking, mental disorders, and suicides, are not uncommon in regions under the impact of large enterprises, such as the municipalities examined in this study. For this reason, adaptations were made to the index to reflect the specificity of the enterprise and the socioeconomic and demographic conditions. Ethnic minorities are significantly affected if aspects of their cultures that interfere with adaptation and survival are not considered.

Because the PPIC area is located on the border between the municipalities of São Gonçalo do Amarante and Caucaia, and because the latter municipality has a sizeable territorial extension with very distinct patterns in population density, well-defined rural and urban areas, and the residence of minority ethnic groups, we chose to study both municipalities with census sectors as units of analysis.

The proximity of the sources of emission and dispersion was defined as 1.6 km. It is noteworthy that the average distance between the census sectors and the sources was 786 m, and the standard deviation was 68.2 m. In 473 census sectors there was no proximity to polluting sources in the two municipalities. The only census sectors that were considered to be close to polluting sources belong to the districts of Catuana and Pecém, in Caucaia and São Gonçalo do Amarante, respectively. The remaining census sectors were not within a 1.6 km radius around the polluting sources of the CIPP. However, this study did not consider other industries that may have arisen or benefited from the construction of the CIPP, and they are not included in the legally delimited area and may be located in other points of Caucaia and São Gonçalo do Amarante. 

Levels of exposure to atmospheric pollution are influenced by the seasons and weather conditions (wind direction and strength, levels of rain and air humidity). The lowest value of the air pollution index was 0.4074, in a census sector in Serrote, São Gonçalo do Amarante, and this does not necessarily refer to the greater distance from the PPIC emitting sources. The contamination of water by coliforms at levels higher than expected occurred exclusively in census sectors of the municipality of São Gonçalo do Amarante.

The fact that the five largest CERI were found in census sectors in the district of Pecém shows that, from an environmental point of view, this is the most affected area in the study area and that this arose because of the PPIC, either due to the proximity of polluting sources or to atmospheric pollution. This district was also noteworthy for containing census sectors where there was water contamination. Between 1991 and 2010, the population of the district of Pecém grew approximately 70.76%, while the people of the municipality grew by approximately 49.87% [1]. In this way, the sudden population growth tends to overburden the supply of services, including housing and sanitation, in a municipality where slightly less than half of the census sectors were classified as rural and, in a district where ¾ of the census sectors were rural. This situation is not only reflected in the Pecém district, but also in its neighbour Taiba, which is economically characterized by, among other things, temporarily housing PPIC workers. However, this district also has limitations in the provision of sanitation, perhaps even more intense than Pecém, as CEARÁ (2013) [1] highlights that in Taíba there is no water supply so that the population is supplied through shallow artesian wells and it does not have a sewage system.

In general, in the study area areas with a low proportion of families were identified. Even in the headquarters district of Caucaia, where there were heterogeneous levels of poverty, low poverty was concentrated in particular census sectors. In our study, social vulnerability was influenced by proximity to the capital of Ceará, being located on the coastal strip or located in the district having the headquarters of municipalities. However, such an indicator has the limitation of, in the conception of Vinhais and Souza (2016) [18], of not being sufficient for economies with high growth rates, as is the case in the current study. Because of this complexity, the income indicator is insufficient because the availability of infrastructure and urban infrastructure is less than demand. Thus, because the government does not meet the urban needs of specific groups, these, in turn, need to supply its own resources. In addition, in a socially heterogeneous country such as Brazil, the purchasing power of a minimum wage varies widely between states. This finding is paralleled by the Superintendency of Socioeconomic Studies of Bahia (SEI, 2008) [19], where it is stated that poverty cannot be conditioned by income. Therefore, it is necessary to consider other factors, including health, education, and housing. Furthermore, Zubinsky [20], when addressing the distribution of poverty in American cities, claimed that the race factor complements the income factor in explaining the distribution of poverty in cities. Thus, the SVI contains certain conceptual proximities with new perceptions about poverty and related conditions.

The main limitations of this study are related to the availability of secondary data of many dimensions because we chose to use tools already applied to assessments of cumulative environmental vulnerability and the level of health. Another limitation of this study is the cross-sectional nature of the analysis presented here allows the examination of geographic or spatial variations. Still, it does not illuminate how these variations unfolded over time. It was necessary to geocode a large amount of data on morbidity (hospitalisation) and vital statistics, including mortality and live births. These data, obtained from multiple information systems, did not have an adequate address record. The challenge of using these secondary data is a tiring but necessary task to highlight the conditions that are influenced by large enterprises.

## 5. Conclusions

Environmental vulnerability is heterogeneously distributed, and the most impoverished areas are also the most vulnerable. The cumulative environmental vulnerability assessment carried out in this investigation supports the hypothesis about the influence of the PPIC in the environment and allowed the delimitation of the space where the socio-environmental effects of this great enterprise are materialised. Vulnerability is considered to be related to both sustainability and natural and environmental risks in the context of climate change, in both social and economic terms. Another approach to assessing vulnerability is health, which is highly relevant in this study. The concept of vulnerability is comprehensive and adapts to each area of knowledge. This study proposed the use of an index to analyse the construct “socio-environmental vulnerability”, integrating social, economic, and urban infrastructure because of the precarious living conditions of the population (work, education, income, sanitation, mobility), and linking this to environmental conditions, health, and public safety.

This study also helps future discussions of the extent of this area of influence, as it was demonstrated that consequences vary according to the proximity of PPIC. For example, the detection and delimitation of the effects observed in the Pecém district in São Gonçalo do Amarante provided scientific evidence for decision-makers to defend the adoption of mitigating measures. Another essential aspect to highlight is that not only the area around the PPIC exhibited a high degree of vulnerability, but also that other areas in the municipality of Caucaia were also vulnerable.

## Data Availability

As Brazilian law establishes, secondary data can be obtained upon request from the Health Department of the State of Ceará.

## References

[1] A L E do Ceará (2013). Cenário Atual do Complexo Industrial e Portuário do Pecém.

[2] (2013). Atlas do Desenvolvimento Humano No Brasil: Base de Dados. http://www.atlasbrasil.org.br/acervo/biblioteca.

[3] Bezerra M.D.G.V., Rigotto R.M., Pessoa V.M., Silva F.V.E.D. (2014). Implicações do desenvolvimento econômico no trabalho, ambiente e saúde em comunidades portuárias no Ceará, Brasil. Ciência Saúde Coletiva.

[4] Ministério do Meio Ambiente (2014). As Implicações da Dinâmica Demográfica dos Países do Bloco BASIC na Agenda de Sustentabilidade.

[5] Corburn J. (2017). Concepts for Studying Urban Environmental Justice. Curr. Environ. Health Rep..

[6] EPA. Environmental Justice. https://www.epa.gov/environmentaljustice.

[7] Adeola F.O., Picou J.S. (2017). Hurricane Katrina-linked environmental injustice: Race, class, and place differentials in attitudes. Disasters.

[8] Sabapathy A., Saksena S., Flachsbart P. (2014). Environmental justice in the context of commuters’ exposure to CO and PM10 in Bangalore, India. J. Expo. Sci. Environ. Epidemiol..

[9] Shi L., Stevens G.D., Lebrun L.A., Faed P., Tsai J. (2008). Enhancing the Measurement of Health Disparities for Vulnerable Populations. J. Public Health Manag. Pract..

[10] Defur P.L., Evans G.W., Hubal E.A.C., Kyle A.D., Morello-Frosch R.A., Williams D.R. (2007). Vulnerability as a Function of Individual and Group Resources in Cumulative Risk Assessment. Environ. Health Perspect..

[11] Penna N.A., Ferreira I.B. (2014). Social and spatial inequalities and areas of vulnerability in the cities. Mercator.

[12] Huang G., London J.K. (2012). Cumulative Environmental Vulnerability and Environmental Justice in California’s San Joaquin Valley. Int. J. Environ. Res. Public Health.

[13] Sadd J.L., Pastor M., Morello-Frosch R., Scoggins J., Jesdale B. (2011). Playing It Safe: Assessing Cumulative Impact and Social Vulnerability through an Environmental Justice Screening Method in the South Coast Air Basin, California. Int. J. Environ. Res. Public Health.

[14] Solomon G.M., Morello-Frosch R., Zeise L., Faust J.B. (2016). Cumulative Environmental Impacts: Science and Policy to Protect Communities. Annu. Rev. Public Health.

[15] Alexeeff G.V., Faust J.B., August L.M., Milanes C., Randles K., Zeise L., Denton J. (2012). A Screening Method for Assessing Cumulative Impacts. Int. J. Environ. Res. Public Health.

[16] Carmo M.E.d., Guizardi F.L. (2018). O conceito de vulnerabilidade e seus sentidos para as políticas públicas de saúde e assistência social. Cad. Saúde Pública.

[17] Silveira M., Fenner A.L.D. (2017). Avaliação de Impactos à Saúde (AIS): Análises e desafios para a Vigilância em Saúde do Brasil. Ciência Saúde Coletiva.

[18] Vinhais H., Souza A. (2006). Pobreza Relativa ou Absoluta? A Linha Híbrida de Pobreza No Brasil.

[19] Santos E.I.d., Carvalho Í.C.S.d., Barreto R.C.S. (2017). Pobreza multidimensional no estado da Bahia: Uma análise espacial a partir dos censos de 2000 e 2010. Rev. Adm. Pública.

[20] Guo Y., Chang S.-S., Sha F., Yip P.S.F. (2018). Poverty concentration in an affluent city: Geographic variation and correlates of neighborhood poverty rates in Hong Kong. PLoS ONE.

